# Decreasing Burden of Malaria in Pregnancy in Malawian Women and Its Relationship to Use of Intermittent Preventive Therapy or Bed Nets

**DOI:** 10.1371/journal.pone.0012012

**Published:** 2010-08-06

**Authors:** Gaoqian Feng, Julie A. Simpson, Ebbie Chaluluka, Malcolm E. Molyneux, Stephen J. Rogerson

**Affiliations:** 1 Department of Medicine (RMH/WH), University of Melbourne, Melbourne, Australia; 2 Centre for Molecular, Environmental, Genetic & Analytic Epidemiology, School of Population Health, University of Melbourne, Melbourne, Australia; 3 Malawi-Liverpool-Wellcome Trust Clinical Research Program, College of Medicine, University of Malawi, Blantyre, Malawi; 4 School of Tropical Medicine, University of Liverpool, Liverpool, United Kingdom; Université Pierre et Marie Curie, France

## Abstract

**Background:**

The World Health Organization recommends insecticidal bednets and intermittent preventive treatment to reduce malaria in pregnancy. Longitudinal data of malaria prevalence and pregnancy outcomes are valuable in gauging the impact of these antimalarial interventions.

**Methodology/Principal Findings:**

We recruited 8,131 women delivering in a single Malawian hospital over 9 years. We recorded demographic data, antenatal prescription of intermittent preventive therapy during pregnancy with sulfadoxine-pyrimethamine and bed net use, and examined finger-prick blood for malaria parasites and hemoglobin concentration. In 4,712 women, we examined placental blood for malaria parasites and recorded the infant's birth weight. Peripheral and placental parasitemia prevalence declined from 23.5% to 5.0% and from 25.2% to 6.8% respectively. Smaller declines in prevalence of low birth weight and anemia were observed. Coverage of intermittent preventive treatment and bednets increased. Number of sulfadoxine-pyrimethamine doses received correlated inversely with placental parasitemia (Odds Ratio (95% CI): 0.79 (0.68, 0.91)), maternal anemia (0.81, (0.73, 0.90)) and low birth weight from 1997–2001 (0.63 (0.53, 0.75)), but not from 2002–2006. Bednet use protected from peripheral parasitemia (0.47, (0.37, 0.60)) and placental parasitemia (0.41, (0.31, 0.54)) and low birth weight (0.75 (0.59, 0.95)) but not anemia throughout the study. Compared to women without nets who did not receive 2-dose sulfadoxine-pyrimethamine, women using nets and receiving 2-dose sulfadoxine-pyrimethamine were less likely to have parasitemia or low birth weight babies. Women receiving 2-dose sulfadoxine-pyrimethamine alone had little evidence of protection whereas bednets alone gave intermediate protection.

**Conclusions/Significance:**

Increased bednet coverage explains changes in parasitemia and birth weight among pregnant women better than sulfadoxine-pyrimethamine use. High bed net coverage, and sulfadoxine-pyrimethamine resistance, may be contributing to its apparent loss of effectiveness.

## Introduction

Malaria in pregnancy is a major public health problem in sub-Saharan Africa, causing significant maternal and infant morbidity and mortality [1], [2], [3], [4]. Recent decreases in pediatric malaria infection [5], [6] and malaria burden [7] may be due to improved access to effective treatments including artemisinin combination therapies, or to increased coverage with insecticide-treated nets (ITN). Globally, the World Malaria Report shows recent declines in malaria cases and deaths [8], [9], possibly reflecting a combination of real progress and improved ascertainment of correct information.

In 1993 Malawi introduced intermittent preventive treatment during pregnancy (IPTp), comprising two treatment doses of sulfadoxine-pyrimethamine (SP) for all pregnant women at Antenatal Clinics. SP has been shown to protect pregnant women from malaria [10], but the spread of SP resistant *P*. *falciparum* [11], [12], [13] raises the importance of investigating the ongoing effectiveness of SP IPTp. Provision of ITNs through health facilities, communities and the private sector has increased population coverage in Malawi [8], but the impact of increased coverage on malaria in pregnancy has not been widely studied.

Using longitudinal data collected between 1997 and 2006 in Blantyre, Malawi, we have investigated the changes in malaria prevalence among pregnant women and their pregnancy outcomes in a single hospital, and how the changes in these outcomes are associated with SP IPTp and bed net coverage.

## Results

### Summary of study population

Between 1997 and 2006, we recruited 8131 pregnant women. Of these women, 4031 were primigravid (49.6%), 4088 were multigravidae (50.3%) and gravidity was not recorded for 12 women (0.1%). The percentage of primigravid women varied between 39.5% in 1997 and 54.0% in 1999; 48.8% of women delivering in 1997–2001 and 50.0% of women delivering from 2002 onwards were primigravidae. Overall, 796 out of 7671 women (10.4%) had peripheral parasitemia, 667 out of 4831 women (13.8%) had placental parasitemia, 2278 out of 7964 women (28.6%) were anemic and 555 out of 4715 (11.8%) newborn infants were LBW.

For women who did not deliver while the study team was present, we were not able to collect information on placental malaria infection or birth weight. However, the characteristics of these women were similar to those of women with delivery data available (Table 1).

**Table 1:** Characteristics of women with and without delivery data available.

	Delivery data	No delivery data
Primigravidae	2254/4712 (47.69%)	1777/3407 (52.36%)
Rainfall over 6 months prior to delivery (mm)^a^	585 (574, 596)	572 (559, 585)
Doses of SP received at ANC		
0	611/4535 (13.5%)	408/3304 (12.3%)
1	1617/4535 (35.7%)	919/3304 (27.8%)
2	1533/4535 (33.8%)	1183/3304 (35.8%)
3+	774/4535 (17.1%)	794/3304 (24.0%)
Hemoglobin (g/dl) b	11.9±1.9	11.8±1.8
Anemia	1295/4593 (28.2%)	983/3371 (29.2%)

a Geometric mean and 95% CI.

b Mean ± S.

### Trends in malaria prevalence and malaria related outcomes from 1997 to 2006

**Figure 1:**
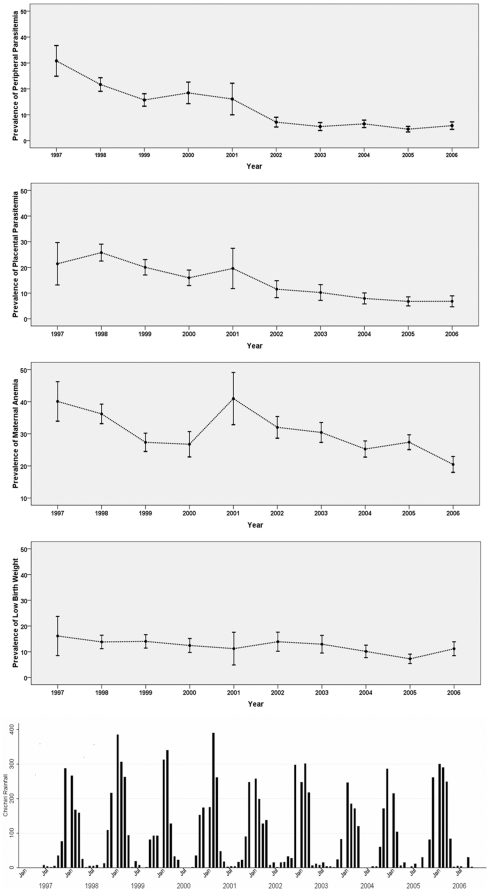
Prevalence of parasitemia, anemia and LBW and monthly rainfall in Chichiri from 1997 to 2006. Lines indicate prevalence and error bars indicate 95% CI, bars indicate accumulated monthly rainfall

Over the study period there were declines in prevalence of peripheral and placental parasitemia, maternal anemia and LBW (Figure 1). The prevalence of peripheral parasitemia dropped from 23.5% during 1997-8 to 5.0% during 2005-6. Prevalence of placental malaria decreased from 25.2% during 1997-8 to 6.8% during 2005-6.

**Figure 2:**
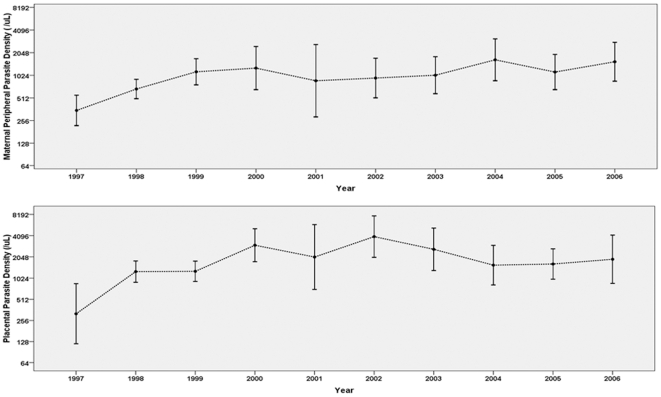
Summary of parasite density among malaria infected women from 1999 to 2006. Lines indicate geometric mean parasite density and error bars indicate 95% CI.

Among malaria infected women, between 1997-8 and 2005-6 peripheral parasite densities among malaria infected women increased from a geometric mean of 568/µl to 1302/µl, and placental parasite density increased from 1091/µl to 1737/µl (Figure 2). Over the same period, the prevalence of maternal anemia decreased from 37.0% to 24.5%; the average hemoglobin concentration increased from 11.5 g/dl to 12.1 g/dl; the prevalence of LBW decreased from 14.1% to 8.9%; and the mean birth weight increased from 2927 g to 3024 g (Figure 1).

There were no significant changes in rainfall patterns over the study period (Figure 1).

After adjusting for gravidity, season and local rainfall, we observed a strong association between year of enrollment and prevalence of malaria and associated pregnancy outcomes (Table 2). The odds of peripheral parasitemia declined by 22% each year, while the odds of placental parasitemia, maternal anemia and LBW decreased by 19%, 6% and 6% per year, respectively.

**Table 2:** Changes in prevalence of parasitemia, anemia and LBW due to time, rainfall, gravidity and season^a^.

	Peripheral parasitemia(n = 7671)		Placental parasitemia(n = 4831)		Maternal anemia(n = 7964)		LBW(n = 4715)	
	OR(95% CI)	P-value	OR(95% CI)	P-value	OR(95% CI)	P-value	OR(95% CI)	P-value
Each year increase	0.78(0.76, 0.80)	<0.001	0.81(0.79, 0.84)	<0.001	0.94(0.93, 0.96)	<0.001	0.94(0.91, 0.97)	<0.001
Two-fold increase in rainfall (mm)^b^	1.03(0.99, 1.09)	0.13	1.08(1.02, 1.14)	0.01	0.97(0.94, 1.00)	0.09	1.15(1.07, 1.22)	<0.001
Multigravidae	0.81(0.70, 0.94)	0.01	0.65(0.55, 0.77)	<0.001	1.15(1.04, 1.26)	0.01	0.53(0.44, 0.64)	<0.001
Dry season	0.59(0.50, 0.68)	<0.001	0.51(0.43, 0.61)	<0.001	0.75(0.68, 0.82)	<0.001	0.83(0.70, 1.00)	0.05

NOTE. –LBW  =  Low Birth Weight; OR  =  Odds Ratio; CI  =  Confidence Interval;

a Mutually adjusted for year of enrollment, rainfall, gravidity and season of enrollment;

b Total rainfall over 6 months prior to delivery date.

### SP IPTp coverage from 1997 to 2006

**Figure 3:**
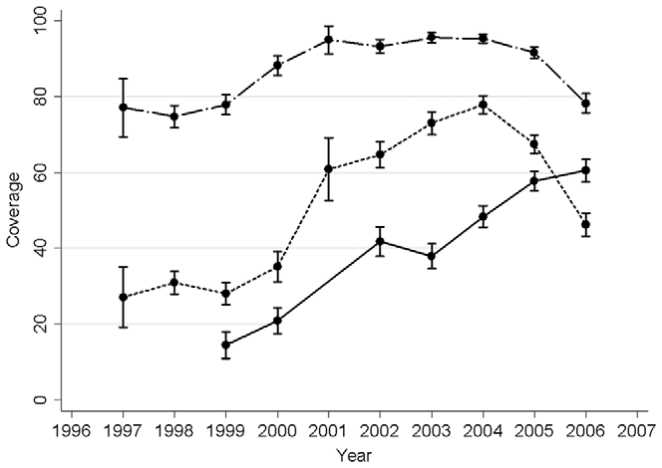
SP IPTp and ITN coverage between 1997 and 2006. Percentage of women receiving the first dose of IPTp (dashed line), second dose of IPTp (dotted line), and women reporting bednet use (solid line). Error bars indicate 95% CI.

The percentage of women receiving at least one dose of SP IPTp (documented on their ANC cards) increased from 77.0% in 1997 to 95.3% in 2004. For the second documented dose, the coverage increased from 27.0% in 1997 to 77.8% in 2004. Coverage decreased in 2005 and 2006 (Figure 3). The number of SP doses recorded, and the number of doses reported to have been taken, were highly correlated (r = 0.84).

### Effect of SP IPTp on prevention of malaria and subsequent outcomes from 1997 to 2006

We previously observed an inverse relationship between number of doses of SP IPTp prescribed and prevalence of malaria infection, anemia and LBW [14]. *P. falciparum* parasites resistant to SP have become highly prevalent in Malawi [15], so we explored whether SP effectiveness has changed over time. After adjusting for season of enrollment, gravidity and rainfall, the relative reduction in the odds for placental parasitemia, hemoglobin and birth weight were modified by year of enrollment. Among women enrolled from 1997–2001, the number of SP IPTp doses was associated with protection against placental parasitemia, maternal anemia and LBW, and with increases in mean maternal hemoglobin levels and birth weights, whereas from 2002 onwards, these protective associations were not found (Table 3).

**Table 3:** Association between doses of SP IPTp treatment and prevalence of parasitemia, anemia and LBW.

	Overall^a^		Enrolled before1^st^ Jan 2002^b^	Enrolled after1^st^ Jan 2002^b^	
	OR^c^ (95% CI)	P-value	OR^c^ (95% CI)	OR^c^ (95% CI)	P-value^e^
Peripheral parasitemia(n = 7671)	0.91(0.84, 1.00)	0.050	0.96(0.84, 1.09)	0.95(0.84, 1.07)	0.941
Placental parasitemia(n = 4831)	0.85(0.77, 0.94)	0.002	0.79(0.68, 0.91)	0.95(0.82, 1.10)	0.062
Maternal anemia(n = 7964)	0.96(0.91, 1.01)	0.134	0.81(0.73, 0.90)	1.00(0.94, 1.07)	0.001
LBW(n = 4715)	0.79(0.72, 0.88)	<0.001	0.63(0.53, 0.75)	0.90(0.78, 1.03)	0.002

	β^d^ (95% CI)	P-value	β^d^ (95% CI)	β^d^ (95% CI)	P-value^e^
Hemoglobin (g/dl)(n = 7964)	0.03(−0.02, 0.07)	0.216	0.16(0.06, 0.26)	0.01(−0.04, 0.07)	0.007
Birth weight (g)(n = 4715)	36.5(21.0, 51.9)	<0.001	74.9(48.2, 101.6)	15.2(−4.5, 34.8)	<0.001

NOTE. –SP  =  Sulfadoxine-Pyrimethamine; IPTp =  Intermittent Preventive Therapy OR  =  Odds Ratio; CI  =  Confidence Interval; LBW  =  Low Birth Weight.

a Adjusted for year and season of delivery, gravidity and 6 months rainfall prior to delivery;

b Adjusted for season of delivery, gravidity and 6 months rainfall prior to delivery;

c Odds Ratio for each additional dose of SP IPTp prescribed through antenatal clinics, up to 3 doses;

d Predicted mean change for each additional dose of SP IPTp prescribed through antenatal clinics, up to 3 doses;

e From likelihood ratio test investigating interaction between SP IPTp treatment and year of enrollment (before/after 1^st^ 2002)

### Bed net coverage in protection against malaria in pregnancy

Information on bed net coverage was not recorded prior to 1999, leaving data available for analyses from 6064 pregnancies. Bed net coverage increased substantially from 14.4% in 1999 to 60.5% in 2006 (Figure 3), and was associated with a decreased odds for peripheral or placental parasitemia and LBW, but not anemia (Table 4). Infants' birth weight was 47 g higher in women using bed nets than in non-users (Table 4). The protective effect of bed nets was not changed between 1999–2002 and 2003–2006 (data not shown).

**Table 4:** Association between bednet usage and prevalence of parasitemia, anemia and LBW^a^.

	Bednet use	
	OR^b^ (95% CI)	P-value
Peripheral parasitemia(n = 5665)	0.47 (0.37, 0.60)	<0.001
Placental parasitemia(n = 3574)	0.41 (0.31, 0.54)	<0.001
Maternal anemia(n = 5925)	0.94 (0.83, 1.06)	0. 29
LBW(n = 3496)	0.75 (0.59, 0.95)	0.02

	β^c^ (95% CI)	P-value
Hemoglobin (g/dl)(n = 5925)	0.07 (−0.02, 0.17)	0.14
Birth weight (g)(n = 3496)	46.8 (13.8, 79.9)	0.005

NOTE. –LBW  =  Low Birth Weight; OR  =  Odds Ratio; CI  =  Confidence Interval;

a Adjusted for year and season of delivery, gravidity and 6 months rainfall prior to delivery.

b Odds Ratio for women using bednet compared to women who did not use bednet.

c Predicted mean change for women using bednet compared to women who did not use bednet.

Among women receiving at least two doses of SP IPTp and using a bed net, the prevalence of peripheral malaria, placental malaria and LBW was significantly decreased in comparison to women without bed nets and receiving one, or no, doses of SP IPTp. Hemoglobin levels were marginally higher in the women who had used bed nets and received optimal dosing of SP IPTp compared with women receiving sub-optimal SP IPTp and not using a bed net. Women with bed nets but with ≤1 dose of SP IPTp showed intermediate levels of protection, whereas women receiving ≥2 SP IPTp doses without nets were not significantly protected from malaria or its consequences (Table 5).

**Table 5:** Association between full dose SP IPTp (more than 2 doses) and bednets and prevalence of malaria, anemia and LBW^a^.

	Bednet only		SP only		Bednet and SP	
	OR (95% CI)^b^	P-value	OR (95% CI)^b^	P-value	OR (95% CI)^b^	P-value
Peripheral parasitemia	0.54(0.38, 0.79)	0.001	0.87(0.67, 1.13)	0.31	0.38(0.27, 0.53)	<0.001
Placental parasitemia	0.52(0.35, 0.75)	0.001	0.98(0.75, 1.28)	0.90	0.33(0.23, 0.49)	<0.001
Maternal anemia	0.86(0.70, 1.04)	0.13	0.90(0.77, 1.06)	0.20	0.88(0.75, 1.05)	0.17
LBW	0.59(0.30,1.16)	0.13	0.77(0.59, 1.03)	0.08	0.57(0.41, 0.79)	0.001

	β (95% CI)^c^	P-value	β^c^ (95% CI) c	P-value	β (95% CI)^c^	P-value
Hemoglobin (g/dl)	0.15(−0.01, 0.31)	0.06	0.11(−0.02, 0.24)	0.10	0.14(0.00, 0.27)	0.05
Birth weight (g)	66.8(16.5, 117.2)	0.009	55.5(14.1, 96.8)	0.009	85.0(40.4, 129.6)	<0.001

NOTE. –SP  =  Sulfadoxine-Pyrimethamine; IPTp =  Intermittent Preventive Therapy OR  =  Odds Ratio; CI  =  Confidence Interval; LBW  =  Low Birth Weight.

a Adjusted for season of delivery, gravidity and 6 months rainfall prior to delivery.

b Odds Ratio relative to women receiving less than 2 doses of SP IPTp and no bed net.

c Predicted mean change from women receiving less than 2 doses of SP IPTp and no bed net.

## Discussion

In this study, we demonstrate marked changes in prevalence of malaria parasitemia among pregnant Malawian women attending a tertiary hospital for delivery over a 10-year period. These changes were associated with concomitant decreases in prevalence of maternal anemia and LBW, important indicators of maternal malaria morbidity. This result is in keeping with declines in malaria illness in young children from East and West Africa, over similar time frames [5], [6].

Together with the decreased prevalence of malaria infection, maternal anemia and LBW, we observed increased coverage with SP IPTp and with bed nets. In other studies in subsets of participating women, we found that the proportion of bed nets that were ITNs increased from 69% in 1997–1999 to 89% in 2001–2006 (SJR, unpublished). Increased coverage with IPTp and ITNs appears to be the likeliest explanation for the changes we have documented. While SP primarily protects the individual woman, ITNs may both protect the individual and (through their mosquito-killing activity) have community-wide effects.

Earlier studies showed that SP use was associated with decreased prevalence of placental malaria, maternal anemia and LBW [14], but coverage was low [14], [16], leading to initiatives to simplify policy and improve access to SP [17]. These resulted in a significant increase in IPTp coverage rates; the reasons underlying recent declines are not known, but may include turnover of ANC staff and unmet needs for ongoing training, and/or logistic difficulties in drug procurement and delivery. Identifying the obstacles to maintaining IPTp coverage at high levels is an important research priority.

The relationship between IPT use and parasitemia, anemia and low birth weight changed over time. Between 1997 and 2001 SP IPTp was associated with decreased placental malaria, maternal anemia and LBW, but these associations were lost from 2002–2006. We considered two possible explanations: increased parasite resistance to SP, and masking of a protective effect of SP by increased ITN coverage or by declining malaria prevalence and malaria-attributable morbidity, for other external reasons. Parasite resistance may compromise the efficacy of SP as treatment for malaria [13], [18]. Studies have demonstrated a high prevalence of SP-resistant *P*. *falciparum* in Malawi [12], [15], [19] associated with SP treatment failure in children and pregnant women [13], [20]. Published data from children and pregnant women in Blantyre over the study period show that “quintuple” mutant parasites with two mutations in the dihydropteroate synthase (*dhps*) gene and three dihydrofolate reductase (*dhfr*) mutations were highly prevalent in Blantyre over the study period [19], [21], but mutations associated with higher level SP resistance such as *dhps* 581[15] or *dhfr* 164 [19] remained rare. It appears unlikely that the loss of SP effectiveness can be ascribed solely to increasing drug resistance.

Increasing bed net coverage (from 14.4% to 60.5%, with increasing proportions of these ITNs) might make SP appear less effective. We found that bed net use was constantly associated with protection against malaria infection and LBW, as described for ITNs [22]. Cochrane analyses show that both IPTp and ITNs increase birth weight, and SP IPTp decreases severe maternal anemia [10], [23], but it is less clear whether the two interventions have additive benefits [22], [24]. When we compared women using bed nets and SP IPTp singly and in combination to those using neither, women receiving both interventions had a lower prevalence of peripheral parasitemia, placental parasitemia and LBW than other women. It may be that administration of SP IPTp was synergistic with bed net use in our population; alternatively, women receiving both interventions may be at lower risk of malaria in pregnancy or its complications owing to differences in variables that we did not measure, such as education or socioeconomic status. We were limited in our ability to examine fully the effects of contemporaneous changes in SP use, bed net use and malaria transmission by our sample size; larger studies from areas with differing rates of SP and ITN use would be needed to resolve this question.

As previously reported [23], [25], we found that bed nets were not protective against anemia, suggesting that the decrease in anemia may have occurred through other mechanisms, or possibly reflects a community-wide change in the impact of malaria on anemia, rather that a direct protective effect of bed nets. This is in contrast to observations in children from Blantyre over a similar period, in whom sleeping under an ITN was associated with significantly higher hemoglobin levels [26].

Our data collection ended in 2006, and in 2007 Malawi adopted artemether-lumefantrine as first line treatment for malaria in children [27], retaining SP for IPTp. Withdrawal of chloroquine first line treatment in Malawi in 1993 was followed by subsequent re-emergence of chloroquine sensitive parasites [28]. Whether withdrawal of SP for treatment will lead to re-emergence of SP-sensitive parasites is unknown, but the widespread use of other antifolates such as cotrimoxazole may inhibit any such process. In South East Asia, parasite genetic markers of sulfadoxine resistance remain at high levels long after SP was replaced in Cambodian antimalarial treatment policy[29].

Changes in rainfall may cause fluctuations in malaria transmission and in the prevalence of parasitemia, but these seemed not to explain the large changes in malaria observed. HIV increases susceptibility to malaria [30], but rates of HIV seroprevalence among women attending our hospital were relatively constant [31], [32], suggesting that changing HIV epidemiology is also not responsible. We know of no changes in hospital use or other host factors to explain the changes seen; there are no data on entomological inoculation rates for Blantyre over the study period. The proportion of malaria-susceptible primigravid women did not change consistently over the time of the study.

In areas of high malaria transmission, pregnancy-specific immunity develops over the first one or two pregnancies. In children, interruption of malaria transmission leads to decreased specific immunity and increased susceptibility to severe disease [33], [34], and it will be important to determine whether the declining prevalence of malaria in pregnancy leads to decreased pregnancy-specific immunity, and an increased risk of severe disease among those women still being infected. In our study, parasite prevalence fell from levels consistent with stable transmission to levels comparable to those reported from areas of low or unstable transmission [4]. This was associated with an increase in the mean parasite density amongst infected women consistent with weakening immune regulation of parasite density. We did not record symptomatology in our participants, but improved control of malaria in pregnancy may result in increasing proportions of malaria-infected pregnant women developing severe or symptomatic disease.

This study does have some potential limitations. Although we recruited more than 8,000 women, these women only count for less than 10% of the total deliveries during the study period. Moreover, the number of study participants included each year varied because of changing availability of staff to recruit study participants; although logistical issues associated with starting a separate study [35], [36] meant that we had limited recruitment in 2001, decreasing the precision of our measurements for that year, the findings from this smaller sample are consistent with overall annual trends observed. Because the study team was only present during normal working hours, we could not collect delivery data from women who delivered out of hours. This missing delivery data could potentially have biased our findings, but we believe this is unlikely, because characteristics of women with and without delivery data were very similar (Table 1). We could not uniquely identify each woman, and women could have been recruited in successive pregnancies leading to potential within-person correlations of outcomes among these women. However, it is unlikely with such a large sample that multiple enrolments would significantly affect our conclusions regarding SP IPTp and bed net use (p-value <0.001 for most analyses).

A recent paper critiqued facility-based surveys as tools to evaluate changing malaria epidemiology [37]. Because we used hospital-based convenience sampling we cannot claim that our data are representative of changing epidemiology in the whole community, but by using the one facility, and the same approach to measurement of all variables throughout the study, we believe changes in measures over time are robust. We documented ANC SP prescription from ANC cards, but also noted a very strong correlation (r = 0.84) between number of SP doses recorded on the ANC cards and doses women self-reported as taking during pregnancy from any source, and only 1.9% of women reported taking fewer doses of SP than were recorded on ANC cards. Because Malawian policy encourages primigravidae and women at risk to deliver at hospital and other women to deliver at health centers, relatively high proportions of primigravidae at higher risk of malaria were recruited, which could lead to an over estimate of the prevalence of malaria. In a separate study of HIV-malaria interactions in pregnancy, we performed malaria microscopy on 3825 different women admitted to the antenatal and delivery wards of the same hospital near term. Nine percent had peripheral blood parasitaemia [36]. This figure is similar to the overall parasite rate we observed, of 10.4%, and to the rate we observed in women recruited to the present study over the same period from 2001–2004, of 6.7%. It is therefore unlikely that our sampling has systematically biased us to collect women with a particularly high prevalence of malaria infection. Because of the demonstrated benefit of IPTp and ITNs, observational studies are the only ethical way to study the relationship between intervention use and outcomes.

It is not clear how representative our data are of Malawi as a whole, or of malaria-endemic countries in sub-Saharan Africa. Most women in our study were urban residents, and may have self-selected to deliver in hospital. Local programmes to improve uptake of SP IPTp [17] or ITNs may have a geographically restricted impact. Changes in malaria prevalence may vary across different regions of a country [38]. Similar longitudinal studies, in a mix of urban and rural areas in Malawi and in other malaria-endemic countries should be undertaken. Such information will be valuable to document possible changes in malaria epidemiology following increased implementation of malaria control and treatment strategies including ITNs, indoor residual spraying and ACTs.

In conclusion, malaria infection declined significantly among pregnant women delivering in Blantyre between 1997 and 2006, and this was accompanied by declines in prevalence of anemia and LBW deliveries. Mean birth weights increased by 97 g, a change of similar magnitude to the reported benefits of effective malaria chemoprophylaxis or IPTp in pregnancy [10], and greater than that reported with use of ITNs [23]. We postulate that differences in the use, and protective efficacy, of IPT or bed nets might explain these changes. Using part of this data set we previously reported that SP use was associated with protection against parasitemia, LBW and anaemia [14], but this association disappeared over the course of the present study. Whilst it is not clear from our data that increasing SP resistance explains this change, there is nevertheless an urgent need to find alternatives to SP for use in IPTp. Bed net use was strongly associated with decreased malaria infection and improved birth weight, underlining the importance of bed nets as a malaria control tool.

Importantly, our data suggest that, among women with good access to health facilities, high rates of coverage with IPTp and ITNs can be achieved, and that these may be associated with significant reductions in the burden of malaria in pregnancy. With an increasing focus on reducing the malaria burden, it will be of great value to discover whether similar reductions can be obtained in other settings, through scale up of these recommended interventions to WHO's target levels.

## Materials and Methods

### Ethics Statement

Ethics approval for this study was given by the Malawi Health Sciences Research Committee and the Research Ethics Committee of the College of Medicine, University of Malawi.

### Study site and population

We recruited women delivering in the maternity unit of Queen Elizabeth Central Hospital in Blantyre, Malawi, an urban hospital hosting 15,000 deliveries annually. Malaria transmission in Blantyre is perennial with a seasonal peak during the rainy season. Primigravidae and at-risk pregnant women are specifically encouraged to deliver in the hospital.

### Data collection

We recruited study participants between July 1997 and August 2006. Data from 1644 women delivering between July 1997 and November 1999 were included in a previous publication [14].

Participants were selected under the same criteria as previously described [14]. Women attending the maternity unit for delivery gave verbal consent for participation. Those who were not in active labor or were unlikely to deliver during working hours were not recruited.

We recorded participants' demographic and clinical data and noted the number of doses of SP prescribed at Antenatal Clinics (ANC) as shown on ANC cards. We separately asked women the number of courses of SP they had received during pregnancy. We used finger prick blood samples to prepare thick blood films for immediate microscopy for maternal malaria parasitemia and to measure hemoglobin using a HemoCue hemoglobinometer (HemoCueAB, Angelholm, Sweden). When women delivered in the presence of the study team, we pricked the cleaned maternal placental surface with a grooved stylet (which collected a droplet of blood in its groove) and used this droplet to make a thick blood film. All thick blood films were examined twice by trained microscopists, counting at least 200 white blood cells, and parasite counts were recorded as the average of the two readings. If results disagreed (positive vs negative, or marked difference in density), a third independent reading was performed, and the two closest results were averaged. An assumed white cell count of 6000/µl was used to convert parasite counts to densities per µl. While study microscopists changed over the study period, the procedures were consistent throughout.

We collected information on bed net use from 1999 to 2006 except for 2001 by asking whether the women were usually sleeping under a bed net. Information on whether the net was insecticide impregnated, or when, was not routinely available. From the Bureau of Meteorology, Malawi, we obtained monthly rainfall data for Chichiri, Blantyre between 1997 and 2006.

### Data analysis

We classified participants as primigravidae (first pregnancy) or multigravidae (second and subsequent pregnancy). Doses of SP IPTp were classified as no SP treatment, 1 dose, 2 doses, or ≥3 doses. For some analyses, we divided year of enrollment into early (1997–2001) and late (2002–2006). Bed net usage was coded as whether or not the woman usually slept under a bed net. We defined anemia as hemoglobin concentration <11 g/dl and low birth weight (LBW) as infant's birth weight <2500 g. Seasons were classified as rainy (November to April) and non-rainy (May to October) based on the delivery date. Monthly rainfall data were summed over the 6 months prior to delivery to represent rainfall exposure during the 2^nd^ and 3^rd^ trimesters. Cumulative rainfall exposure was not normally distributed, and, therefore, the variable was log_2_ transformed before analysis.

The exposure variables of interest were SP IPTp (0, 1, 2 or ≥3 doses) and bed net use. Outcome variables were peripheral parasitemia (detection of parasites in peripheral blood), placental parasitemia (detection of parasites in placental blood), maternal hemoglobin concentration at delivery and anemia, infant's birth weight and LBW. Maternal gravidity, season of enrollment and rainfall were considered as confounding factors. We performed multivariable logistic regression, adjusting for the confounders described above, to investigate how the prevalence of maternal peripheral parasitemia, placental parasitemia, maternal anemia and LBW changed over time and how these outcomes varied with SP IPTp and bed net use. We also analyzed the impact of SP IPTp and bed net use on maternal hemoglobin level and birth weight using multiple linear regression. To explore whether the SP IPTp protection effect was modified over the study period, an interaction term between SP IPTp and year of enrolment (before versus after 1^st^ January 2002, the mid-point of study period) was fitted, and tested using the likelihood ratio test. To investigate the combined effect of SP IPTp and bed net use we included a variable with the following categories: ≥2 doses of SP IPTp and bed net use; ≥2 doses of SP IPTp only; sub-optimal (≤1 dose) SP IPTp and bed net use; and no bed net and sub-optimal SP IPTp during pregnancy. All statistical analyses were performed using Stata 9.2 (StataCorp, College Station, TX, USA).
